# Ischemic priapism caused by self intracavernous injection of tadalafil

**DOI:** 10.1002/iju5.12695

**Published:** 2024-01-27

**Authors:** Naoki Wada, Tsubasa Hatakeyama, Haruka Takagi, Ryoken Tsunekawa, Shin Kobayashi, Masaya Nagabuchi, Takeya Kitta, Hidehiro Kakizaki

**Affiliations:** ^1^ Department of Renal and Urologic Surgery Asahikawa Medical University Asahikawa Japan

**Keywords:** priapism, proximal shunt, tadalafil

## Abstract

**Introduction:**

We present a case of ischemic priapism caused by self intracavernous injection of tadalafil.

**Case presentation:**

A 77‐year‐old man developed priapism due to self‐injection of tadalafil into the corpus cavernosum. He presented to our hospital 2 days after the development of priapism and severe penile pain. The blood gas analysis of the corpus cavernosum revealed ischemic priapism. At first, we performed percutaneous distal shunt (T‐shunt) and cavernosal irrigation, resulting in slight improvement of penile tumescence. Several hours later, penile tumescence and severe pain reappeared. Bilateral proximal (corpora‐spongiosal) shunt was performed under anesthesia again. Penile tumescence was slowly and gradually relieved. His erectile function was declined.

**Conclusion:**

We experienced a case of priapism due to self intracavernous administration of tadalafil who needed a proximal shunt to relieve the severe penile pain. This case report may serve as a warning for physicians and patients not to use phosphodiesterase 5 inhibitor inappropriately.

**Abbreviations & Acronyms**
BGA: blood gas analysis
ED: erectile dysfunction
PDE5: phosphodiesterase 5

**Keynote message**

Ischemic priapism is diagnosed by cavernous blood gas analysis, and prompt aspiration of blood or shunt surgery is recommended to prevent irreversible fibrosis of the corpus cavernosum. This is the first case report of ischemic priapism caused by self intracavernous injection of tadalafil. This is a warning for physicians and patients not to use PDE5 inhibitor in a wrong way.

## Introduction

Priapism is characterized by a persistent penile erection despite sexual interest or desire and can be divided into two types; ischemic and non‐ischemic priapism. Ischemic priapism is a major andrological emergency. It may damage erectile tissue and can induce ED. While it is rare, there have been reports of PDE5 inhibitors being associated with priapism. Herein, we present a case of ischemic priapism caused by self intracavernous injection of tadalafil.

## Case report

**Fig. 1:**
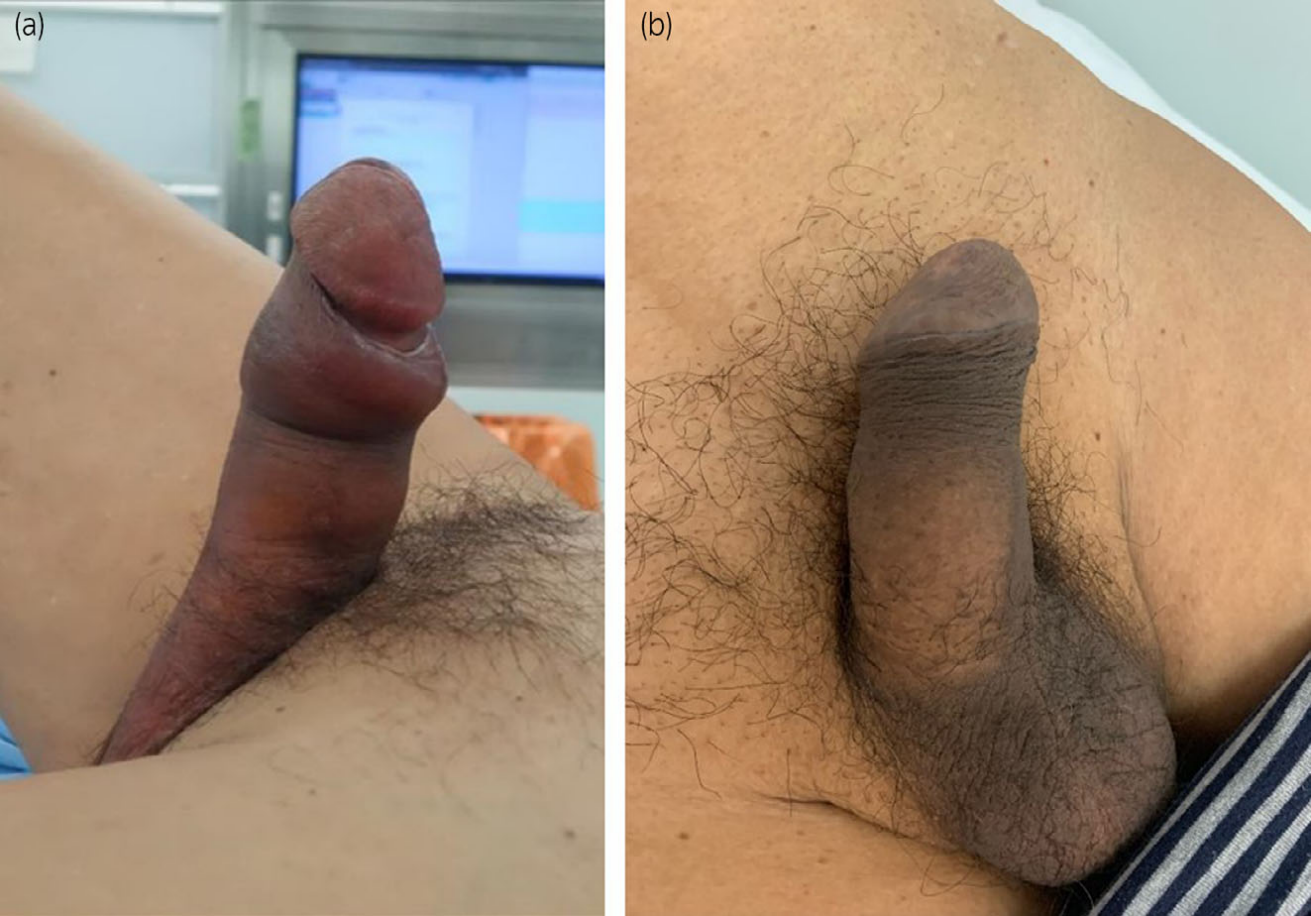
(a) Rigid penis before distal shunt. (b) Penile tumescence was relived 1 month after the surgeries.

A 77‐year‐old man developed priapism after self‐injection of tadalafil into the corpus cavernosum. He originally had a sufficient erectile function for sexual intercourse with or without oral PDE5 inhibitor. He crushed the tadalafil tablet prescribed from his physician himself and dissolved it in commercially available purified water. It seemed that he injected tadalafil into the corpus cavernosum using a syringe that he received from his physician to gain further erectile strength. Since severe penile pain appeared after the continuous erection for 2 days, he consulted the previous clinic. At the previous clinic, the BGA of the corpus cavernosum (pH: 6.88, pO_2_: 4.7 and pCO_2_: 69.5 mmHg) revealed ischemic priapism. Aspiration of the cavernosal blood and injection of phenylephrine hydrochloride and norepinephrine were performed; however, both procedures were not effective (Fig. 1a). He was referred to our department for further treatment.

**Fig. 2:**
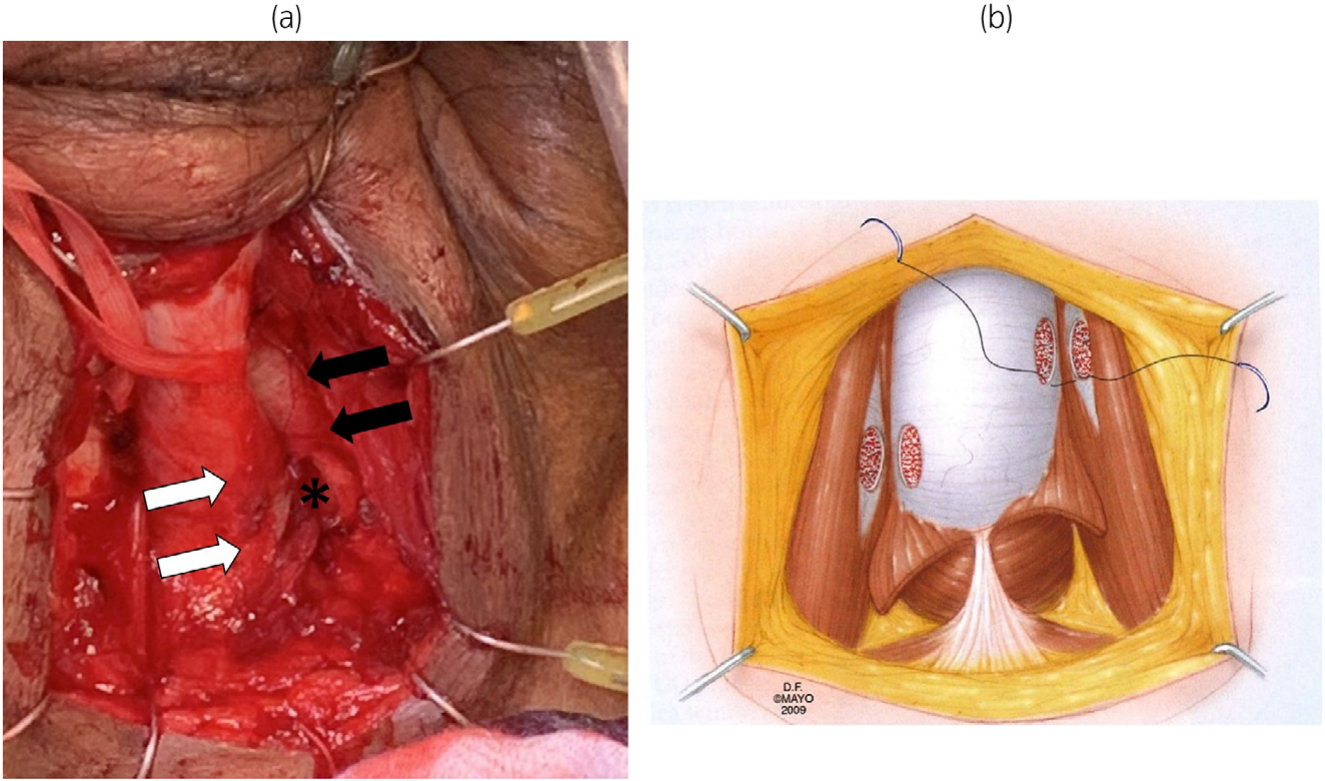
(a) A communication (*) between the corpus cavernosum (black arrows) and the corpus spongiosum at the level of bulbar urethra (white arrows). (b) A illustration of proximal shunt from Campbell‐Walsh‐Wein UROLOGY 12th Edition.

He had no history of taking antipsychotics. Complete blood count showed no abnormalities that suggested any blood disorders. We performed percutaneous distal shunt (T‐shunt) and cavernosal irrigation under general anesthesia. Those resulted in the slight improvement of BGA of corpus cavernosum (pH: 7.42, pO_2_: 60.6, and pCO_2_: 22.3 mmHg) and penile tumescence. However, several hours later, penile tumescence and pain reappeared. Then, we performed bilateral corpora‐spongiosal shunt (Sacher method) under general anesthesia (Fig. 2a,b).^1^

**Fig. 3:**
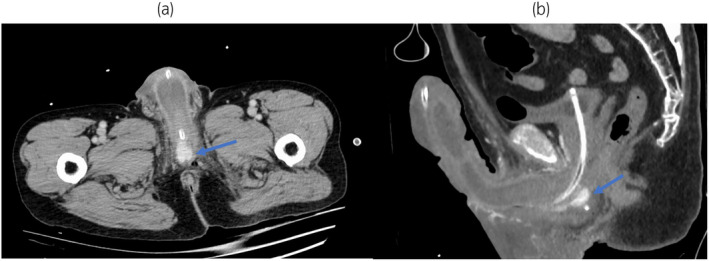
The corpus spongiosum at the level of bulbar urethra is enhanced (arrow) in the arterial phase suggesting the blood flow from the corpus cavernosum via proximal shunt. (a) axial plane, (b) sagittal plane.

Although penile erection had been persistent, contrast‐enhanced computed tomography showed the shunt flow from the corpus cavernosum to the corpus spongiosum (Fig. 3a,b). His penile tumescence was slowly and gradually relieved, whereas a little rigidity of the penis remained 1 month after the surgeries (Fig. 1b). His erectile function is impaired after the surgeries.

## Discussion

This is a case report of ischemic priapism caused by self intracavernous injection of tadalafil, requiring a proximal shunt to relieve the severe penile pain and resulting in impaired erectile function. This case report may serve as a warning to physicians and patients to avoid incorrect use of PDE5 inhibitor.

Ischemic priapism, which is also called veno‐occlusive priapism or low flow priapism, is a persistent erection marked by rigidity of the corpus cavernosum in the absence of an arterial cavernous inpour. It consists of an imbalance of vasoregulatory mechanisms, inducing a penile compartment syndrome which is characterized by hypoxia. Treatment should be commenced as promptly as possible to prevent irreversible fibrosis of the corpus cavernosum.

Rahoui *et al*. published their data of 40 patients with ischemic priapism. The causes of priapism included sickle cell disease in 26 (65%), malignant hemopathy in 4 (10%), idiopathic in 4 (10%), neuroleptic drugs in 3 (7.5%), and sildenafil in 3 (7.5%).^2^ According to the World Health Organization global database of individual case safety reports (VigiBase) between 1983 and 2021, a total of 31 827 individual case safety reports were identified relating to PDE5 inhibitors for sexual dysfunction.^3^ Among them, the total number of priapism events was 258 (0.8%). Rezaee *et al*. found 411 cases of drug‐induced priapism secondary to PDE5 inhibitors reported to the Food and Drug Administration since 1998.^4^ More cases of priapism induced by second‐generation antipsychotics (n = 1065) and the antidepressant/sleep aid trazodone (n = 817) were reported. Thus, priapism caused by PDE5 inhibitors is not a common situation. A post‐marketing surveillance study for safety by Eli Lilly indicated that the risk of priapism due to tadalafil was <1 in 10 000.^5^ In the midst of limited case reports of priapism by PDE5 inhibitors, especially tadalafil, this is the first report of priapism caused by intracavernous injection of tadalafil.

The present case was diagnosed as ischemic priapism by the corporal BGA followed by corporal aspiration and phenylephrine injection in a previous hospital. After ischemic priapism is diagnosed, treatment should proceed in stages using a combination of corporal aspiration and phenylephrine injection with or without irrigation. The present case was referred to our department because the penile pain and rigidity did not improve by the first‐line treatment. We performed the distal corpora‐glanular shunt as the surgical management. However, because the distal shunt failed to improve penile pain and tumescence, we had to resort to the proximal shunt as the next surgical option. According to the AUA guidelines, patients should be counseled that there is no adequate evidence to quantify the benefit of performing a proximal shunt, and several clinical considerations should be made in deciding on whether a proximal shunt is appropriate and should be performed.^6^ We believe that the proximal shunt significantly alleviated the penile pain in the present case.

In the present case, the rigidity of the penis remained for a while, and the recovery of erectile function is not observed. King *et al*. presented a case of ischemic priapism due to oral tadalafil administration.^7^ Also in that case, the erection initially rebounded but gradually receded after proximal shunting. Mean half‐life of tadalafil mean is about 17.5 h. Tadalafil is expected to be present and possibly pharmacologically active in the penile tissues. Munarriz et al. demonstrated that reperfusion for ischemic priapism causes erectile tissue injury due to oxidative stress.^8^ Bennett *et al*. reported that patients with priapism sustaining for more than 12 h noted a decrease in erectile rigidity.^9^ Of patients with priapism sustaining for 12–36 h, 44%–78% were able to have functional erection. No patient with priapism over 36 h had spontaneous erection. In the present case, it took nearly 2 days from the onset of priapism to receive treatment. Thus, unfortunately in the present case, it seems that prolonged ischemic priapism caused by local injection of tadalafil resulted in irreversible ED.

## Conclusions

Tadalafil is an effective oral medication for ED. We experienced a case of priapism due to the incorrect use of tadalafil, requiring a proximal shunt. This case report may serve as a warning for physicians and patients to use as PDE5 inhibitor correctly.

## Author contributions

Naoki Wada: Conceptualization; data curation; investigation; writing – original draft. Tsubasa Hatakeyama: Conceptualization; data curation; investigation; writing – original draft. Haruka Takagi: Data curation; investigation. Ryoken Tsunekawa: Data curation; investigation. Shin Kobayashi: Data curation; investigation. Masaya Nagabuchi: Data curation; investigation. Takeya Kitta: Data curation; investigation. Hidehiro Kakizaki: Conceptualization; supervision; writing – review and editing.

## Conflict of interest

The authors declare no conflict of interest.

## Approval of the research protocol by an Institutional Reviewer Board

Not applicable.

## Informed consent

Written informed consent was obtained from the patient.

## Registry and the Registration No. of the study/trial

Not applicable.
